# Nano- and Micro-Polymer Fibers for Smart Actuation: Fabrication Methods and Applications—A Review

**DOI:** 10.3390/gels12060495

**Published:** 2026-06-02

**Authors:** Tarek Dayyoub, Kabiru Haruna, Mohannad Mayyas

**Affiliations:** 1 Interdisciplinary Research Center for Advanced Materials (IRC-AM), King Fahd University of Petroleum and Minerals (KFUPM), Dhahran 31261, Saudi Arabia; kabiru.haruna@kfupm.edu.sa (K.H.); mohannad.mayyas@kfupm.edu.sa (M.M.); 2 Department of Materials Science and Engineering, College of Chemicals and Materials, King Fahd University of Petroleum and Minerals, Dhahran 31261, Saudi Arabia

**Keywords:** polymer fibers, electrospinning, extrusion, shape memory fibers, electroactive fibers, hydrogel fibers

## Abstract

Polymeric fibers represent a vital class of functional materials due to their versatile properties, such as wide availability, low cost, recyclability, biodegradability, and excellent mechanical and chemical stability. Polymer fibers can be fabricated at both micro- and nanoscale dimensions using a variety of processing techniques. This review provides a comprehensive overview of the principal methods employed for polymer fiber preparation, including electrospinning, melt and solution blowing, dry and wet spinning, template synthesis, phase separation, and self-assembly. The technical principles, as well as the advantages and limitations, of each technique are systematically discussed. The review also explores polymeric fibers as smart materials for actuation applications. Particular focus is given to stimulus-responsive fiber systems such as shape memory fibers, hydrogel fibers, liquid crystal fibers, and electroactive polymers. Overall, this review establishes a coherent framework linking polymer fiber fabrication strategies with structure–property–function relationships, offering practical guidance for material selection and accelerating the development of next-generation smart polymer fibers for advanced actuation and multifunctional applications.

## 1. Introduction

Polymers are widely prevalent in modern materials and technologies, making them indispensable in today’s contemporary society. However, the fabrication of polymers and polymer composites with enhanced properties, such as high mechanical strength, chemical resistance, and/or thermal stability, remains a critical challenge for scientists and engineers worldwide. Owing to their distinctive structural and functional characteristics, polymeric fibers are regarded as one of the most important classes of polymeric materials [1]. Polymeric fibers may be naturally found or artificially manufactured. In this context, numerous natural fibers exist, both organic and inorganic, such as silk, wool, cotton, jute, and sisal. On the other hand, a wide range of synthetic fibers can be fabricated, like polypropylene, polyethylene, polyamides, polyethyleneterephthalate (PET), polyacrylonitrile (PAN), polytetrafluoroethylene (PTFE), aramid, and others [2,3]. Natural fibers offer numerous advantages, including wide availability, a low cost, recyclability, non-toxicity, biodegradability, bio-renewability, and a low carbon footprint. In addition, they exhibit favorable mechanical properties, such as strength, toughness, stiffness, and a light weight [4]. These fibers, however, often suffer from limitations in durability and interfacial bonding strength [5]. Synthetic polymeric fibers can be produced from a wide range of monomers as well as from recycled plastic [6,7]. Their key characteristics include ease of dispersion within composite matrices and chemical inertness [8]. However, synthetic fibers often exhibit relatively low elastic moduli and thermal resistance, along with poor interfacial bonding when incorporated into polymer composites [9,10].

From a size-scale perspective, polymeric fibers are generally categorized as microfibers or nanofibers. Nanofibers, with diameters on the order of ~100 nm, exhibit significantly higher surface-area-to-volume ratios, better mechanical properties (strength, stiffness), improved filler–matrix interactions in composites, and more uniform and porous morphologies compared to microfibers. In contrast, microfibers typically range from 1 to 100 µm in diameter, are comparatively thicker and less structurally intricate, and are widely employed in conventional textile applications [11]. It is important to mention that fiber diameters vary from tens of nanometers to a few micrometers depending on the technique and polymers employed [12]. For instance, polyacrylonitrile (PAN) typically produces smooth nanofibers, typically measuring about 200–300 nm in diameter [13]; polyvinyl alcohol (PVA) is able to create extremely thin, eco-friendly fibers, with certain accounts mentioning diameters as small as around 50 nm through bubble electrospinning [14]; polycaprolactone (PCL) commonly generates nanofibers with very small diameters, typically in the <500 nm range, often between 100 and 300 nm when conditions are optimal [15]; poly(methyl methacrylate) (PMMA) can be manufactured into fibers ranging from 0.8 to 2.4 µm based on various conditions [16]. Here, it is important to note that nanofibers typically exhibit enhanced stiffness and tensile strength due to nanoscale effects, often leading to more uniform mechanical behavior when incorporated into composite materials. In contrast, microfibers display more conventional mechanical characteristics and, when used as fillers, may result in uneven hardness. Furthermore, nanofibers can be fabricated with high porosity and a consistent distribution within polymer matrices, enabling the formation of complex and well-interconnected structures. Microfibers, however, typically exhibit uneven shapes with jagged edges when utilized as fillers, resulting in a less even distribution in the composite architecture [17].

**Figure 1:**
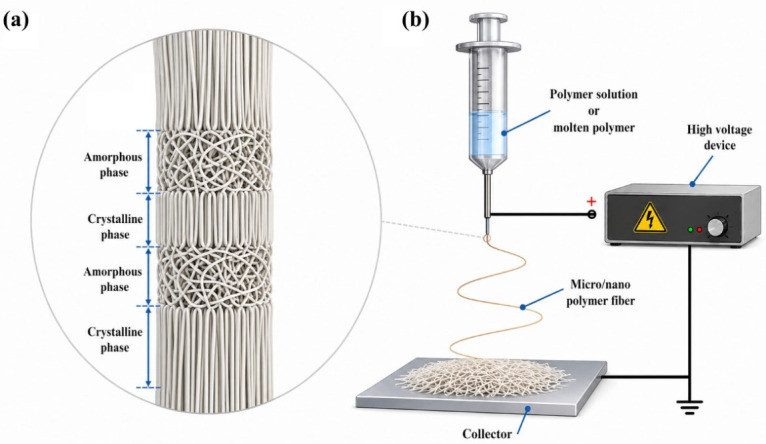
(**a**) Molecular structure of semi-crystalline polymer fibers; (**b**) electrospinning of polymer fibers.

Polymeric fibers exhibit a hierarchical arrangement (Figure 1a). At the molecular level, extended polymer chains are structured into ordered crystalline regions and disordered amorphous ones. At the microscopic scale, a fiber consists of aligned crystalline and amorphous phases, where the crystalline regions mainly impart tensile strength, thermal stability, and chemical resistance, whereas the amorphous regions contribute to properties like flexibility and softness. At a macroscopic scale, the structure of the fiber is characterized by its surface morphology and cross-sectional geometry, which differ significantly among various fiber types [18,19]. Here, it should be noted that the macromolecules in the fiber structure can be oriented during the manufacturing and processing stages, which gives them their unique properties. In particular, both the degree of crystallinity and the orientation ratio are regarded as crucial for a fiber’s properties.

Owing to their light weight, good mechanical properties, and excellent chemical and thermal resistance, polymeric fibers have found widespread application across the textile, construction, automotive, and aerospace sectors. They are used in protective gear like bulletproof vests and fire-resistant suits, in reinforced materials such as composites for aircraft and sports equipment, and in everyday products like clothing and ropes [20,21,22]. Moreover, polymeric fibers are increasingly utilized in medical applications, such as orthopedic implants and tissue engineering scaffolds, owing to their non-toxicity and biocompatibility [23,24].

In this review, a comprehensive and structured overview of polymer fiber preparation techniques is provided, including electrospinning, extrusion or melt blowing, solution blowing, and dry and wet spinning, with a critical focus on their processing principles, key parameters, and inherent advantages and limitations. This review consolidates the fundamental working principles and key processing parameters of polymer fiber preparation techniques to support the informed selection of suitable fabrication methods. Additionally, the review highlights the emerging role of polymeric fibers as smart materials in actuation applications, classifying them according to their activation mechanisms, such as shape memory, hydrogel, liquid crystal, and electroactive polymeric fibers, thereby establishing a unified framework to support future research and technological development in advanced functional fiber systems.

## 2. Preparation of Nano- and Micro-Polymeric Fibers

Nano- and micro-polymer fibers can be produced through different techniques, with electrospinning being the most popular due to its capability to create thin fibers from a polymer solution when subjected to an electric field. Other methods include melt blowing, where heated air is utilized to stretch the polymer melt into filaments, as well as template synthesis, phase separation, solution blowing, and wet/dry spinning. The choice of fabrication technique is primarily governed by the desired fiber diameter, polymer characteristics, and scale of production.

### 2.1. Electrospinning

Electrospinning is a commonly employed technique for producing nano- and microfibers. It entails projecting a fine stream of a polymer solution into an electric field, leading the substance to be pulled and elongated into fibers while being deposited onto a collector. It is a flexible and fairly straightforward technique, able to generate fibers from a range of substances, such as polymers, ceramics, metals, and composites. Electrospinning began in 1934, when Anton Formhals secured patents for various electrospinning devices, after that in 1964 Geoffrey Ingram Taylor presented the idea of the Taylor cone, establishing the basic principles of electrospinning mechanics [25]. In the first decade of this era, the creation of rotating drum collectors resulted in progress like axially aligned electrospinning, providing better control over the microstructural arrangement of the polymer fibers, especially nano ones. This advancement enabled applications that needed directional mechanical strength, improved filtration efficiency, and optimized scaffolds for tissue engineering [26,27]. The primary advancement of the electrospinning method was the roll-to-roll approach in the late 2010s, resulting in a significant breakthrough for large-scale industrial manufacturing [28,29]. This method addressed scalability issues by facilitating ongoing and high-volume fiber production. Furthermore, recent improvements in roll-to-roll systems have further boosted the consistency, efficiency, and reliability of fiber production, closing the gap between research in labs and large-scale industrial use [30,31]. Here, it is important to emphasize that the primary factors for this method are enhanced voltage regulation, the collector configuration, the material choice, and solution refinement.

Electrospinning is an electrohydrodynamic technique whereby a liquid droplet is charged to create a jet, which is then stretched and elongated to form fibers; see Figure 1b. The primary elements consist of a high-voltage power source, a syringe pump, a spinneret, and a conductive collector [32,33]. In the electrospinning method, either a direct current (DC) or alternating current (AC) can be used as a power source. In electrospinning, the liquid is pushed out from the spinneret, creating a pendant droplet due to surface tension. When electrified, the electrostatic repulsion between surface charges of the same sign shapes the droplet into a Taylor cone, resulting in the ejection of a charged jet. The jet first stretches in a straight path and then experiences intense whipping movements due to bending instabilities. As the jet is elongated into thinner diameters, it hardens rapidly, resulting in the placement of solid fibers on the stationary collector. Typically, the electrospinning procedure is segmented into four sequential stages: (a) charging the liquid droplet and creating a Taylor cone or conical jet; (b) the straight-line elongation of the charged jet; (c) jet thinning under an electric field, leading to the emergence of electrical bending instability; and (d) the solidification and gathering of the jet into a solid fiber at a grounded collector (connected to an earth ground) in order to establish a powerful, steady electric field between the nozzle (high voltage) and the collector [34,35,36]. It is important to highlight that, in electrospinning, electrostatic forces generate high-voltage electric fields that surpass polymer surface tension, resulting in the formation of a Taylor cone and the ejection of a charged jet. These forces stretch the polymer fiber via whipping instability and direct it to a grounded collector to create nano-/microfibers. In other terms, a Taylor cone arises when a high-voltage power source is connected to a needle filled with a polymer solution, leading to charge buildup on the liquid’s surface. The resultant electrostatic push surpasses surface tension, transforming the hemispherical droplet into a conical form referred to as a Taylor cone. When the critical voltage is attained, the charged jet is expelled from the apex of the Taylor cone toward the grounded collector. The charged, liquid stream undergoes bending instability due to the electrostatic repulsion present within the stream [37].

Electrospinning parameters can be categorized into machine parameters (voltage, flow rate, etc.), solution parameters (polymer concentration, viscosity, etc.), and ambient parameters (temperature, humidity, etc.). These factors significantly influence the final fiber’s diameter, morphology, and mechanical properties. Properly adjusting these parameters is crucial for producing high-quality, smooth, and bead-free fibers for specific applications [38]. The applied voltage in electrospinning typically falls within the range of 5 to 50 kV, depending on the material and desired fiber properties [39,40]. The effects of the flow rate are highly dependent on the applied voltage. This means that a higher voltage can help to overcome the effects of a higher flow rate, but it can also lead to bead formation if not balanced correctly. On the other hand, a higher flow rate may be acceptable with a more concentrated or viscous solution, which has a greater ability to stretch. In addition, using a smaller needle can help to reduce the effects of a high flow rate by creating a smaller initial droplet [41,42]. The polymer concentration is a critical parameter in electrospinning that affects fiber formation, bead formation, and the fiber diameter, often requiring a specific range for optimal results. In this context, an overly low concentration can lead to beads, while an overly high concentration may result in an unstable jet or no fiber formation. It is essential to find the correct concentration for the polymer and solvent being used in order to produce smooth, bead-free fibers with the desired characteristics [36,43]. Viscosity is a critical electrospinning parameter that influences fiber formation and diameters, with an ideal range for most applications of typically between 1 and 1000 centipoise (cP). A solution that is too low in viscosity will result in electrospraying (droplets) instead of fibers, while a solution that is too high will be impossible to extrude. The polymer concentration is the primary way to control the viscosity, and it is crucial to achieve the correct viscosity for bead-free fiber production [44]. Moreover, the temperature is also considered a critical electrospinning parameter that affects solvent evaporation, viscosity, and the fiber diameter. For a given system, increasing the temperature can decrease the solution viscosity and lead to smaller fiber diameters up to an optimal point, beyond which faster solvent evaporation can prevent proper jet stretching and increase the fiber diameter. Finding the optimal temperature depends on the specific polymer and solvent used [45]. In addition, humidity affects the fiber morphology, diameter, porosity, and collection efficiency, with the specific outcome depending on the polymer’s properties, such as its hydrophobicity and solvent miscibility. Generally, low humidity can lead to high charge loss and poor fiber formation, while increased humidity can induce surface pore formation in some polymers and decrease the fiber diameter due to faster evaporation and charge loss in others. An optimal humidity level, often around 40–50%, is needed to balance these effects and achieve the desired fiber characteristics, although this varies between materials [46,47].

A major advantage of electrospinning is its capacity to function at room temperature, rendering it a highly adaptable and energy-saving method for producing fibers from heat-sensitive materials. It generates dry fibers in one step, removing the need for lengthy post-processing [36]. Moreover, generating adaptable fibers with adjustable porosity and an easy, affordable configuration can also be considered an advantage of the electrospinning method [40]. Nonetheless, drawbacks consist of scalability challenges, possible solvent toxicity, needle blockage, and difficulties in managing the final 3D shape and guaranteeing consistency, Moreover, a major drawback of the method is that not every sought-after material can be utilized [48]. Table 1 summarizes the preparation methods for polymer fibers, their sizes, the types of used polymers, and the advantages and disadvantages.

### 2.2. Extrusion or Melt Blowing

**Figure 2:**
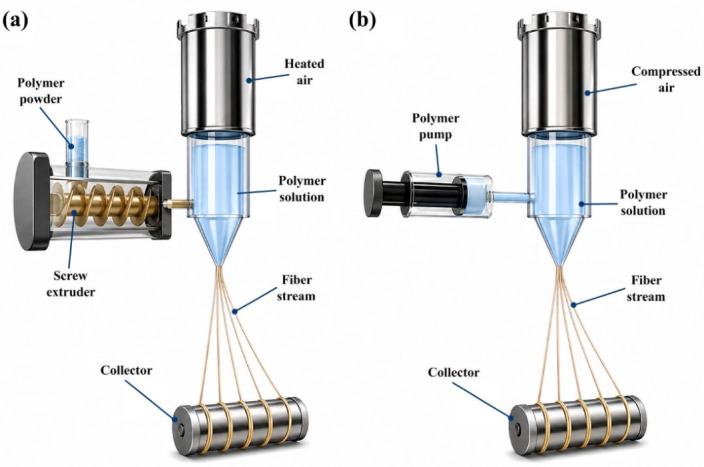
(**a**) Melt blowing process; (**b**) solution blowing process.

Extrusion is a technique that is used to shape polymers into forms such as fibers, films, tubes, sheets, etc., whereas melt blowing is a particular, single-step method that employs high-speed air to stretch molten polymers into extremely fine fibers for producing nonwoven fabrics. In melt blowing, thermoplastic polymers are extruded through a die, and rapid attenuation is performed by a hot air stream to create fine fibers (Figure 2a). The attenuated fibers are subsequently positioned on a collector screen to create a self-bonded, fine-fibered mat. The integration of fiber-to-fiber bonding and fiber entanglement ensures the adequate cohesion of the web, allowing it to be used without further bonding. However, the fibers prepared using melt blowing technology range in diameter from 2 to 5 μm [49,50]. While the polymer is extruded through a linear die into converging streams of heated air, the high-speed air diminishes the fibers. The identical air currents transport the fibers to a collector. As the fibers reach the collector, they are cooled, creating entanglements, and bonding occurs at the points where the fibers touch, resulting in a unified nonwoven web. Typically, a vacuum is used at the collector to aid in separating air from the polymer fabric [51].

Significant efforts have been made to create nanofibers via the melt blowing technique by designing and developing modular dies featuring smaller holes and a greater number of holes, enabling the cost-effective production of submicron fibers. A modular die is composed of stacked plates, where one plate features an inlet for molten polymer and heated air inlets, in contrast to the conventional melt-blown die that uses linear holes and air knives to facilitate process air from both sides. The plates are arranged to establish channels that form a route for material flow, with one plate featuring an outlet functioning as a spinneret orifice for the extrusion of polymers. A modular die functions at a low throughput for each hole and, therefore, at lower melt pressures for fiber extrusion. The molten polymer is pushed at a comparatively low flow rate through the orifice of the modular die to create submicron fibers [51].

The melt blowing process depends on several parameters, such as the polymer flow rate, melt temperature, polymer properties, air velocity and temperature, uniform air distribution, die-to-collector distance, collector speed, die design, vacuum level, etc. The polymer flow rate, defined as the volume or mass of polymer extruded through the die at each unit of time, is a vital factor in managing fiber characteristics, and it differs from the polymer’s melt flow index (MFI) [52]. A polymer with a lower MFI needs elevated temperatures and/or increased pressure to attain a specific flow rate, whereas a polymer with a higher MFI will flow more effortlessly. In melt blowing, the melt temperature is regarded as a vital factor that affects the characteristics of the resulting fiber and fabric. Standard melting temperatures vary based on the particular polymer utilized, and they are frequently established between 230 and 360 °C [53]. Nonetheless, an improper melt temperature may result in flaws in the finished nonwoven fabric, including slots, fly, and roping. Additionally, the temperature impacts the viscosity and flow properties of the polymer, subsequently affecting the fiber fineness produced. For uses demanding resistance to high temperatures, polymers with elevated melting points and thermal stability are necessary [53]. Melt viscosity is considered an important characteristic that influences the quality and attributes of the final nonwoven fabric. It influences the fiber diameter, strength, and consistency of the end web and can be regulated by modifying the polymer’s molecular weight and process temperature. Increased viscosity, resulting from a greater molecular weight, results in a thicker fiber diameter, whereas elevated processing temperatures reduce the viscosity, facilitating the extrusion of the polymer [54]. It is well known that the viscosity is directly affected by the polymer’s molecular weight. In simpler terms, polymers with a higher molecular weight exhibit increased viscosity and entanglement, resulting in stronger fibers but also a larger fiber diameter. The selection of the polymer variety and its molecular weight distribution is thus a crucial element in attaining the desired characteristics of the product. Moreover, raising the die temperature decreases the viscosity by diminishing the polymer chain’s flow resistance. This can be utilized to refine the melt flow rate and modify the procedure to accommodate various polymers and intended results [55]. The temperature of the hot air in melt blowing usually ranges from 230 to 360 °C and is essential in drawing the polymer melt into thin fibers. The precise temperature relies on the type of polymer utilized, as low temperatures result in insufficient melting while high temperatures lead to thermal degradation [56]. This means that the hot air’s temperature must be regulated by passing compressed air through a heat exchange unit, like an electric or gas-fired furnace, to achieve the target temperature. It should be noted that, in melt blowing, the hot air pressure generally falls between 60 and 75 kPa for the best outcomes, while the air supply equipment delivers approximately 70–90 kPa. The pressure is managed by a Roots blower or air compressor and is essential for the fiber thickness and web configuration, with increased pressures frequently resulting in thinner fibers [57].

In addition, in melt blowing, the distance from the die to the collector is regarded as a critical parameter that greatly influences the properties of the resulting nonwoven fabric, such as the density, strength, fiber diameter, and pore structure. A shorter distance leads to a denser, firmer web with improved uniformity and barrier characteristics, whereas a longer distance results in a bulkier, softer, and less strong web. The ideal distance from the die to the collector depends on the intended application and is utilized alongside other process variables, such as the air pressure, to manage fiber production [58,59]. This distance influences the amount of time that the fibers have to cool prior to reaching the collector. Reduced distances result in hotter fibers, resulting in increased self-bonding.

In melt blowing, the speed of the collector also greatly influences the properties of the final nonwoven mat, including its porosity, density, and mechanical traits. Boosting the collector speed typically raises the mat’s porosity and lowers its density by separating the fibers more, yet it may also enhance the elastic modulus and ultimate strength of the mat [60]. An increased collector speed leads to the fibers covering a broader area as they move further before being deposited, resulting in a more spacious arrangement, which in turn leads to a decrease in mat density and an increase in material porosity. Another key parameter in melt blowing is the die geometry, which is defined by the slot die and the swirl die. Essential factors for the slot die consist of the slot width, slot angle, and nose piece width, which affect the fiber diameter, whipping action, and attenuation. Various geometries are engineered to manage the airflow and polymer resistance, resulting in differences in fiber creation, movement, and the overall quality of the nonwoven web [61,62]. In this context, the drag force from the air jet reduces the polymer stream into thin fibers. This means that the geometry of the die has a direct impact on the efficiency of this drag force. Furthermore, the shape affects the movement of the fiber, including the whipping action seen in the slot die method. Moreover, factors such as the slot width and the internal manifold configuration of the die are essential in guaranteeing even fiber distribution throughout the width of the nonwoven fabric.

The melt blowing technique enables the production of a significant quantity of material, rendering this method a rapid, economical means of fiber production. It is a solvent-free, high-yield process that creates fine fibers with a wide range of fiber diameters, from the nano to micro size, yielding lightweight, porous fabrics with an extensive surface area [63]. These characteristics render these produced fibers ideal for applications such as filtration, absorption, and insulation, with possibilities for enhanced functionality via techniques like bicomponent and electret melt blowing [64] (Table 1). On the other hand, the drawbacks of melt blowing consist of the significant upfront capital expenditure, the requirement for the accurate management of delicate process variables, and difficulties in creating consistent, high-quality nonwovens. Elevated temperatures may lead to polymer breakdown and possible inhalation hazards from small fibers. Moreover, numerous traditional melt-blown polymers are non-biodegradable and may present an ecological threat [63,65].

### 2.3. Solution Blowing

Solution blow spinning is a fiber production technique that utilizes two parallel concentric fluid streams: a polymer dissolved in a volatile solvent and a pressurized gas that circulates around the polymer solution, forming fibers that are laid down in the direction of gas movement [66,67]. Typically, a solution blowing arrangement includes a compressed gas source to provide the carrier gas and a syringe pump for the polymer solution. The two streams can be straightforwardly combined into a simple, easily produced device or created with a commercially available airbrush [68,69]. In this approach, a polymer is dissolved in a volatile solvent to lower its viscosity and form a spinning solution. Subsequently, the mixture is injected through an inner nozzle as a high-speed gas, typically air, passes through an outer concentric nozzle. This flow generates a low-pressure area around the polymer stream, which, when paired with shearing forces from the high-velocity gas, stretches the solution into a slender jet. Subsequently, as the polymer jet moves toward the collector, the solvent quickly evaporates because of the high gas speed and extensive surface area, resulting in solid polymer fibers (Figure 2b).

The parameters for solution blowing encompass solution characteristics, including the polymer concentration, viscosity, solvent type, and conductivity; they also cover process conditions like the gas pressure, gas flow rate, solution flow rate, nozzle size, and distance from the nozzle to the collector and even environmental variables such as the temperature and humidity. These factors together affect the fiber’s diameter, structure, and mechanical characteristics [70,71]. In more detail, the polymer concentration is viewed as an essential factor influencing the viscosity and fiber development. This means that a concentration that is either too low or too high can result in inferior fiber quality, whereas a moderate range is usually ideal for consistent fiber production. Additionally, the type of solvent significantly affects its volatility, conductivity, and surface tension, which in turn impacts the process and the properties of the fibers. Additionally, the viscosity has a direct connection to the polymer concentration, and it needs to remain within an appropriate range for the solution to stay stable and create a continuous jet [66,70]. Conversely, increased air pressure may reduce the fiber diameter by elongating the polymer jet; however, excessive pressure can cause jet instability. Additionally, the flow rate of the gas impacts the solvent evaporation speed and polymer jet stretching, thereby affecting the fiber structure and production rate. Furthermore, the flow rate of the solution can be modified to regulate the production rate and is typically optimized alongside other factors, such as the pressure [66,72]. Here, it should be noted that the temperature and humidity can affect the solvent evaporation rates.

Solution blowing provides high production speeds and has a low cost, making it ideal for large-scale production. It enables the accurate regulation of the fiber diameter and form through the modulation of the air pressure and polymer concentration, along with the advantage of immediate, on-site deposition. This method is likewise adaptable, allowing for the development of multifunctional materials such as core–shell or three-dimensional fibrous structures [67,73]. Furthermore, solution blowing allows for the direct application of fibers onto a specific area at the location of use, eliminating the need for additional drying or collection processes. This technique can be utilized to produce micro- and nanofibers from various materials, such as polymers, ceramics, and nanocomposites. One of the key features is that, in contrast to electrospinning, which employs a high voltage, solution blowing utilizes a high-speed gas, thus rendering it a safer method. On the other hand, the primary drawbacks of solution blowing for polymer fibers are irregularity and misalignment, as the turbulent airflow results in varying fiber diameters and arbitrary orientations. The procedure is also restricted by the types of polymers and solvents permitted, favoring quick-drying solvents and those with reduced viscosities. Moreover, solution blowing may face challenges such as regular nozzle blockages, complicated setup demands, and increased production expenses, restricting its use in industrial applications relative to alternative techniques [66,74]. In addition, reduced polymer concentrations or elevated surface tension may result in beading rather than consistent fibers. The formation of beads can also be caused by solvent evaporation that occurs either too quickly or too slowly [70]. It is important to highlight that the turbulent airflow generated by the high-velocity gas stream can lead to fibers with varying diameters and unpredictable orientations, presenting challenges for applications that demand high uniformity or alignment. Moreover, obtaining the best outcomes frequently necessitates intricate nozzle configurations and the accurate management of various factors, such as the gas pressure and nozzle shape [67] (Table 1).

### 2.4. Dry Spinning

**Figure 3:**
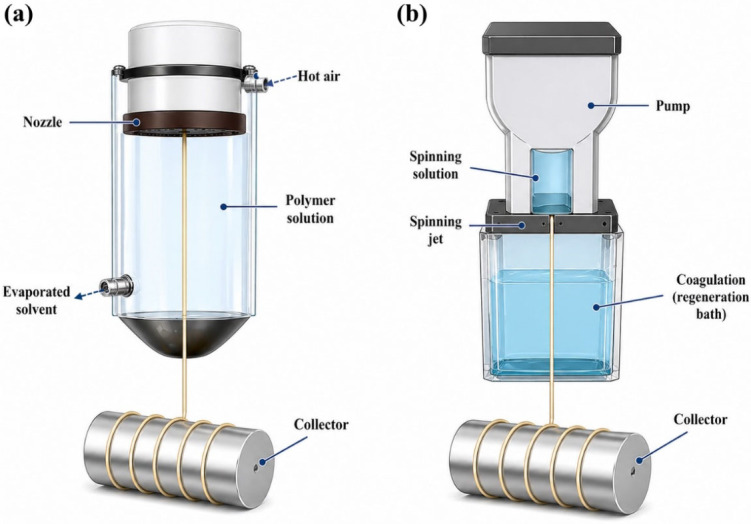
(**a**) Dry spinning process; (**b**) wet spinning process.

The dry spinning method is applied to polymers that do not melt but instead degrade when heated. Dry spinning is necessary for polymers that have a melt temperature at or near their thermal degradation temperature, so they must be dissolved in a solvent to be formed into fibers. In dry spinning, the solvent dissolves the polymer before it is extruded. As fibers pass through the spinneret, the solvent evaporates with heated air. This method can be utilized for creating fibers like acetate, acrylic, etc. [75]. This process consists of several stages (Figure 3a): (a) dissolving the polymer in a solvent that evaporates easily to formulate a dope solution, which is subsequently filtered to eliminate impurities; (b) the extrusion of the solution by forcing it through a spinneret, which has many tiny holes, into a drying tower; (c) the evaporation of the solvent using hot air circulating through the tower; (d) the solidification of the polymer into continuous filaments; (e) the subsequent gathering of the solid fibers on a take-up wheel or winder; (f) the recovery of the solvent from the air within the drying tower via condensation or absorption and its recycling. The main factors in the dry spinning process for polymer fibers include heat transfer, mass transfer, and the mechanical forces exerted on the filament. These are strongly dependent on the characteristics of the solvent and polymer, the rate at which the solvent evaporates from the solution, the temperature and speed of the airflow, and the stretching or drawing of the fiber once it leaves the spinneret [76]. It is important to mention that elevated temperatures boost the evaporation rate of the solvent, whereas the speed of the air stream influences the mass transfer of solvent vapor away from the fiber’s surface and the aerodynamic forces acting on the fiber. Furthermore, both the quantity and dimensions of the holes in the spinneret influence the number and thickness of the filaments.

Dry spinning benefits heat-sensitive polymers by enabling the creation of high-quality fibers from solutions instead of melts. Additional advantages encompass quicker production speeds, easier post-spinning processes, and adaptability in altering the spinning parameters. Additionally, the spinning parameters in this approach can be easily adjusted, and the procedure is quite adaptable. It is also worth mentioning that, in relation to wet spinning, it can accommodate greater polymer concentrations and attain faster spinning rates [77]. The primary drawbacks of dry spinning include the significant expenses associated with substantial investment and energy consumption, as well as environmental and safety issues stemming from the use of hazardous or combustible solvents. It is a more gradual process, necessitates an extra post-spinning stage to eliminate the solvent, and struggles to create fibers with an exact cross-section [78] (Table 1).

### 2.5. Wet Spinning

Wet spinning is a critical fiber formation technique that is particularly suited for polymers that cannot be processed via melt spinning due to thermal degradation or infusibility [79,80]. This method involves dissolving a polymer in a suitable solvent to create a spinning dope, which is then extruded through a spinneret into a coagulation bath containing a non-solvent. The non-solvent induces phase separation, causing the polymer to solidify and form continuous fibers [80,81]. The fundamental principle lies in controlled polymer precipitation, where the diffusion rates of the solvent out of the polymer solution and the non-solvent into the polymer solution dictate the fiber morphology and properties [82].

The wet spinning process is predicated on the principle of non-solvent-induced phase separation (NIPS) [83]. A polymer solution, referred to as the spinning dope, is extruded into a coagulation bath. This bath contains a non-solvent for the polymer but is miscible with the polymer’s solvent. As the polymer solution comes into contact with the non-solvent, the solvent diffuses out of the nascent fiber, and the non-solvent diffuses in. This exchange causes the polymer to precipitate and solidify, forming a fiber structure [81]. The kinetics of this mass transfer process are crucial in determining the fiber’s microstructure, porosity, and mechanical properties [84]. For instance, a rapid exchange rate typically leads to a denser outer skin and a more porous core, while slower exchange can result in a more uniform structure. In a specialized variant, namely dry-jet wet spinning, an air gap is introduced between the spinneret and the coagulation bath [85]. This air gap allows for some solvent evaporation and initial stretching of the polymer jet before it enters the coagulation bath, which can influence the fiber’s orientation and ultimately its mechanical strength [86]. The free-fall spinning method, a type of dry-jet wet spinning, uses gravity alone to deform the polymer solution and nascent hollow fiber, without external tension [85].

Several parameters significantly influence the outcome of the wet spinning process, affecting the fiber morphology, mechanical properties, and overall performance. The dope solution characteristics play a fundamental role, as the polymer concentration and intrinsic viscosity determine the chain entanglement density, spinnability, and final fiber properties [87]. For example, the dope extrusion pressure is a critical parameter in polysulfone hollow-fiber membranes [88], while, in poly(1,4-phenylene-1,3,4-oxadiazole) (POD) fiber spinning, careful regulation of the intrinsic viscosity and temperature is essential for stable extrusion [86]. The solvent type strongly affects the polymer solubility, solution rheology, and diffusion kinetics during coagulation. The spinning conditions are equally critical, as the spinneret diameter and geometry control the initial jet size and shape, with a diameter of 0.4 mm reported for cellulose nanofiber spinning [89], while the spinning rate and stretching speed before or during coagulation strongly influence the molecular orientation, fiber diameter, and mechanical strength [90]. In wet electrospinning, additional stretching induced by an electric field enables the production of ultrafine fibers. The coagulation bath parameters are decisive, as the coagulant type and concentration govern solvent–non-solvent exchange and polymer precipitation. Finally, post-treatment steps such as drawing and stretching after coagulation markedly improve the mechanical properties by aligning polymer chains [87], as evidenced by high-quality poly(acrylonitrile-co-itaconic acid) nascent fibers produced at high draw ratios during dry-jet wet spinning [91], while washing and drying remove residual solvents and non-solvents and consolidate the final fiber structure.

The wet spinning technique is mild and needs a low processing temperature, suitable for natural polymers such as chitosan and collagen, and it provides the ability to adjust the mechanical properties of the final fiber through post-drawing or other tension-related processes. It is especially beneficial for uses such as biomedical scaffolds and the formation of intricate fibrous structures [92,93]. Moreover, it is a favored technique for manufacturing materials such as rayon and is utilized for developing 3D scaffolds with interconnected porous structures for tissue engineering, in addition to constructing intricate 3D forms through direct printing. On the other hand, wet spinning has drawbacks such as slower production rates than melt or dry spinning, the expensive and complicated process of solvent recovery and disposal, and the requirement for extensive post-spinning procedures, such as washing to eliminate contaminants. Moreover, the procedure can take time, and certain polymers might need several baths to achieve full solvent elimination [94,95]. In addition, certain polymers may exhibit differences in domain size and distribution, impacting their overall characteristics [96,97] (Table 1).

### 2.6. Template Synthesis

The synthesis of polymer fibers using a template entails utilizing an existing structure, such as a microporous membrane or self-assembled micelles, to direct the polymerization process. This technique limits the synthesis to produce fibers with diameters defined by the template’s openings, typically resulting in improved characteristics, such as increased conductivity. Methods involve employing membranes as a template and sol–gel electrospinning succeeded by pyrolysis, which utilizes a polymer template that is subsequently decomposed [98,99].

The core principle involves the infiltration of monomer solutions or polymer precursors into the pores or channels of a chosen template, followed by polymerization or solidification within these confined spaces [100,101]. After the material is formed, the template is subsequently removed, leaving behind a polymeric structure that mirrors the template’s morphology [102,103]. This approach allows for the creation of various one-dimensional (1D) structures, such as nanofibers, nanorods, and porous carbon fibers, which are challenging to achieve with other synthesis routes [104,105]. For instance, polyimide hybrid nanofiber aerogels can be constructed using a template-anchored strategy to enhance the mechanical properties [106].

It should be emphasized that several key parameters must be taken into consideration. Initially, factors such as the monomer concentration, molecular weight, solvent selection, and viscosity are regarded as highly significant. The proportions of the monomer and template influence the eventual molecular weight and structure of the polymer. Additionally, a greater polymer molecular weight typically results in denser fibers, particularly in methods such as electrospinning. Furthermore, the solvent needs to adequately dissolve the polymer, possess suitable volatility for evaporation, and retain appropriate viscosity and surface tension for jet creation. Additionally, factors like the temperature, the duration of the reaction, the concentration of the initiator, and the molar ratios of the template, monomer, and crosslinking agents are vital. It is crucial to recognize that the specific polymerization method employed (e.g., photopolymerization, free radical polymerization) and its related conditions, such as the UV wavelength and temperature, are essential factors. Third, the particular type of template and the polymerization technique employed also greatly affect the end products. The template’s structure can affect the tacticity (stereoregularity) of the resulting polymer, forming a complementary structure. Additionally, the template may affect the polymerization rate and the kinetics equation of the reaction [38,107,108,109].

Template synthesis enables the accurate management of polymer fiber characteristics, such as the diameter and morphology, facilitating the development of complex structures. In addition, it serves as a framework enabling the precise regulation of the final fiber’s dimensions, form, and overall architecture. This allows the production of monodisperse fibers that have uniform diameters and lengths. It is beneficial for producing specialized forms such as hollow tubes with distinct inner and outer surfaces, which are valuable for uses like drug delivery. This approach can result in high-quality fibers with consistent dimensions and can be employed to create a diverse range of materials through various templates. Certain template techniques, especially those utilizing accessible porous membranes, are viewed as a potentially low-cost approach for producing various types of microtubules [110,111]. On the other hand, the primary drawbacks of template synthesis for polymer fibers include the challenge of extracting the template without harming the fibers, the restricted fiber length, and the possibility of template aggregation, which compromises the quality of the imprint. Removing a template can be difficult, necessitating particular procedures and possibly resulting in structural failure or harm to the final item. Moreover, template synthesis is unsuitable for creating very long, continuous fibers, and the procedure may be complicated by the low quality of template imprinting, particularly at elevated concentrations [38,112]. However, the main challenge is finding a cost-effective, safe, and stable template (Table 1).

### 2.7. Phase Separation

In polymer fibers, phase separation occurs when a polymer solution divides into two separate phases: one is rich in polymer, and the other is rich in solvent. This distinction results in various fiber shapes, including pores or bi-continuous formations, which are subsequently hardened into the ultimate fiber configuration. The phenomenon can be triggered by altering the temperature (thermally induced phase separation, TIPS), introducing a non-solvent (non-solvent-induced phase separation, NIPS), or subjecting the solution to vapor (vapor-induced phase separation, VIPS) [113,114].

In polymer fibers, phase separation occurs by destabilizing a uniform polymer solution due to external factors such as a temperature shift (e.g., cooling below a specific threshold, referred to as the glass transition temperature (T_g_)) or the addition of a non-solvent (e.g., vapor-induced or non-solvent-induced phase separation), leading it to divide into a phase rich in polymer and a phase rich in solvent, as mentioned earlier. Changes in temperature or the addition of a non-solvent can initiate this process, resulting in a porous structure where the polymer-rich phase solidifies into a fibrous matrix while the solvent-rich phase is eliminated. The solvent-heavy phase is subsequently eliminated using methods such as solvent evaporation or non-solvent substitution. This results in a porous polymer fiber possessing a large surface area and the intended pore configuration [115,116].

Thermally induced phase separation (TIPS) involves forming a homogeneous polymer solution at an elevated temperature by blending a polymer with a high-boiling-point, low-molecular-weight diluent [117]. This solution is then formed into the desired shape, such as a hollow fiber, and subsequently cooled at a controlled rate or quenched to induce phase separation. The key to TIPS is the temperature-dependent miscibility of the polymer and diluent. As the temperature decreases, the system crosses a phase boundary, typically leading to liquid–liquid (L–L) demixing or solid–liquid (S–L) demixing (polymer crystallization) [118,119]. The cooling rate is a critical factor, as rapid cooling can lead to vitrification, whereas slower cooling allows for more extensive phase separation and crystal growth [117]. The morphologies of membranes prepared by TIPS can be precisely controlled by managing the thermodynamic and kinetic aspects of phase separation [120].

Non-solvent-induced phase separation (NIPS) is another common method, whereby a polymer solution is brought into contact with a non-solvent, causing the polymer to precipitate and form a porous structure. This technique is widely used in preparing membranes for various separation processes [121,122]. The process involves the exchange of a solvent from the polymer solution with a non-solvent from an external coagulation bath, leading to a reduction in polymer solubility and subsequent phase separation [113]. The kinetics of phase separation in NIPS are influenced by the diffusion rates of the solvent and non-solvent and the thermodynamic compatibility between the polymer, solvent, and non-solvent [123]. Recent advances in NIPS include novel methods like using hydrogen gas formation from a gas-producing agent within the polymer solution to directly spin hollow-fiber membranes [124].

Hybrid or combined phase separation techniques, such as non-solvent thermally induced phase separation (N-TIPS), leverage aspects of both TIPS and NIPS [125]. N-TIPS involves the use of a non-solvent as part of the thermal phase separation process, often leading to macroporous membranes with narrow pore size distributions [126]. For instance, poly(vinylidene fluoride) (PVDF) hollow-fiber membranes have been prepared by N-TIPS, where the simultaneous occurrence of TIPS and NIPS influenced the membrane morphology and performance [126].

Phase separation benefits polymer fibers by facilitating precise control over the fiber structure and porosity, resulting in increased surface areas for improved performance, as well as allowing for the integration of sensitive or bioactive materials. Major advantages encompass the capacity to produce complex porous designs, customize material characteristics via process management, and employ comparatively gentle conditions, rendering it appropriate for diverse uses such as membranes, scaffolds, and energy capture [126,127,128]. The drawbacks of phase separation in polymer fiber production include possible shrinkage and stress accumulation and the limited management of the pore dimensions and fiber continuity, as well as limitations on the types of polymers and solvent/non-solvent combinations that can be employed. Achieving consistent fiber structures can be challenging, and the procedure may require a considerable amount of time, particularly when it includes several steps, such as thermal induction [128,129,130]. The precipitation process may result in considerable shrinking, potentially reducing the final fiber’s size and porosity. Additionally, considerable contraction may generate stress in the structure, possibly resulting in buckling or various defects. Moreover, the method is confined to polymers that dissolve in the selected solvent and do not dissolve in the non-solvent, making it challenging to properly separate polymers and fillers [131] (Table 1).

### 2.8. Self-Assembly

Self-assembly, a process wherein molecules spontaneously organize into structured arrangements without external guidance, is a crucial technique in the preparation of polymer fibers, offering distinct advantages and limitations in materials engineering [132,133]. This phenomenon is driven by molecular recognition and non-covalent interactions, such as hydrogen bonding, van der Waals forces, and electrostatic interactions, ultimately leading to thermodynamically stable structures [134,135].

The fundamental principle governing self-assembly in polymer fiber formation is the spontaneous organization of individual polymer chains or monomers into ordered, higher-level structures through specific, often weak, non-covalent interactions [136]. This contrasts with traditional synthesis methods that rely on strong covalent bonds and external energy input [137]. For polymers, this often involves hierarchical assembly, moving from molecular interactions to supramolecular structures and eventually to macroscopic fiber morphologies [138].

Molecular recognition plays a critical role, ensuring that specific building blocks interact in a predefined manner to form the desired structures [139,140]. For instance, host–guest recognition motifs are extensively utilized to construct supramolecular polymers with varied topologies and stimulus-responsiveness [141,142]. Dynamic covalent bonds, in conjunction with non-covalent interactions, can also be employed to achieve controlled covalent self-assembly, leading to more stable structures compared to purely non-covalent methods [143,144].

One prominent method for polymer fiber preparation through self-assembly is crystallization-driven self-assembly (CDSA) [145,146]. This technique leverages the crystallization of a core-forming block within amphiphilic block copolymers to direct the formation of well-defined, often fibrous, nanostructures. The sequential CDSA of triblock copolymers, such as poly(p-tert-butylstyrene)-b-polyisoprene-b-poly(3-hexylthiophene) (PtBS-b-PI-b-P3HT), can produce P3HT-based Janus fibers with controlled lengths [145]. Another strategy is polymerization-induced self-assembly (PISA), which combines polymerization with self-assembly to create polymer-based biohybrid nanostructures (PBBNs) and polymeric nanomaterials with inverse morphologies [147,148]. PISA offers advantages in terms of efficiency, versatility, simplicity, reproducibility, and high nanoparticle yields compared to traditional self-assembly techniques. It enables the direct synthesis of block copolymer assemblies during polymerization, which can then form fiber-like structures. Electrostatic interactions are also harnessed, as demonstrated in the self-assembly of diazonium-modified boron nitride nanosheets and glass fibers. In this process, negatively charged functionalized nanosheets and positively charged modified glass fibers are mixed, leading to self-assembly around the glass fiber due to electrostatic attraction [149].

Several parameters can be meticulously controlled to tailor the self-assembly process for polymer fiber preparation, beginning with the polymer concentration, as a minimum critical concentration for net assembly is required to initiate polymer formation, particularly for dynamically unstable filaments [150]. Solvent quality plays a decisive role by modulating non-covalent interactions and the polymer conformation, thereby directly influencing assembly outcomes [151,152]. The temperature plays a vital role in the thermodynamics and kinetics of self-assembly [153]. The effect of supercooling is also relevant, where increased local concentrations of polymers can induce self-assembly during freezing [153]. The pH and ionic strength strongly affect the polymer charge and conformation, especially for polyelectrolytes, thereby regulating electrostatic interactions and assembly pathways [154]. Flow conditions under continuous processing offer enhanced control over molecular and nanoscale architectures by regulating mixing, temperature gradients, and residence times, often outperforming batch processes in terms of structural precision [155]. The molecular curvature and topography, such as those found in saddle-shaped carpyridines, strongly influence one-dimensional supramolecular polymer formation through π–π interactions [156]. Shear forces can also be exploited, as shear-thinning behavior enables the reversible formation of polymeric nanofiber hydrogels, providing practical processing routes where fragmentation would otherwise be irreversible [157]. Finally, the presence of modifiers, including functional groups or nanoparticles, can direct self-assembly behavior, exemplified by the molecular self-assembly of nano-silver/triethylenetetramine onto graphite fiber surfaces, which allows precise control of the interfacial structure and mechanical performance in composite systems [158].

Self-assembly provides benefits for polymer fibers, such as the creation of intricate shapes and layered structures, greater recyclability than continuous fiber production, and the capacity to develop dynamic or self-repairing materials. It also enables accurate regulation over characteristics and the creation of materials with great stability, such as resilient cellulose membranes, as well as easier processing, particularly with shorter fibers [159,160]. On the other hand, the primary drawbacks of self-assembly include insufficient control over the ultimate structure, like the fiber orientation and size, which may result in inadequate stability and the emergence of unwanted aggregates. Additional disadvantages include the complicated and sluggish process that may incur high expenses and lead to reduced productivity [159,160,161] (Table 1).

**Table 1:** Preparation methods for polymer fibers, their sizes, the types of used polymers, and the advantages and disadvantages.

Polymer Fiber Preparation Method	Types of Used Polymers	Typical Diameter	Advantages	Disadvantages	References
Electrospinning	Collagen, chitosan, PVA, PAN, PVDF, PCL	100 nm to 5 µm	- High surface-area-to-volume ratio - Control of tunable properties - Versatility - Low cost - Simple setup - Scalable	- Scalability challenges - Solvent toxicity - Needle clogging - Structure and control limitations - Beading - Repeatability issues - Material limitations	[38,39,40,41,42,43,44,45,46,47,48]
Melt blowing	Thermoplastics, such as PP, PE, PET	Typically 1–10 µm	- High throughput - Fine fiber production - Solvent-free operation - High surface area - High porosity - Versatility - Functional properties	- High capital investment - Complex parameter control - Fiber uniformity issues - Risk of fiber degradation - Potential for “roping” defects	[52,53,54,55,56,57,58,59,60,61,62,63,64,65]
Solution blowing	PEO, PVDF, EVA, PVP, PLA	100 nm to 15 µm	- High production rate - Low cost - Precise control - Direct deposition - Versatility - Material flexibility - Safety	- Polymer and solvent constraints - Bead formation - Lack of uniformity and alignment - Nozzle clogging - Complex setup - High production costs - Limited scalability	[66,67,68,69,70,71,72,73,74]
Dry spinning	PU, PVC, PAN, CA	Typically 10 to 50 µm	- Appropriate for polymers sensitive to heat - High-quality fibers - Quicker manufacturing rate - Easier procedure after spinning - Adaptable procedure - Thin denier strands	- Significant investment and energy expenses - Reduced manufacturing speed - Challenges with staple fibers - Exact cross-section challenge - Post-spinning operations - Environmental and safety concerns associated with solvents	[75,76,77,78]
Wet spinning	Cellulose, PVA, acrylic, PU	25–50 µm	- Suitable for heat-sensitive and natural polymers - Allows intricate fiber designs - Enables functionalization - Tailorable mechanical characteristics - Adaptable for particular uses	- Reduced production speed - Extensive post-spinning procedures - Problems with solvents - Complicated solvent retrieval - Possibility of contaminants - Danger of heat damage	[92,93,94,95,96,97]
Template synthesis	PLA, PS, PC, PMMA, PVP, PANI, PPy	30 nm to 10 µm	- Accurate regulation of shape and size - Formation of intricate frameworks - Divided surfaces - Adaptability - Possibility for expansion - Accurate regulation of shape and size	- Fiber damage - Structural failure - Unproductive elimination - Restricted fiber length - Template compilation - Complexity of processes - Expenses and reliability	[110,111,112]
Phase separation	PLA, PVA, PAN, PAAM, PLGA, PGA, PCL, PEEK, PVDF	50–500 nm	- Accurate regulation of shape and pore structure - Elevated surface area - Gentle processing parameters - Affordability and straightforwardness - Improved capabilities - Enables the integration of additional materials	- Reduction - Accumulation of stress - Selection of polymer and solvent - Ongoing fiber manufacturing - Unhurried kinetics - Labor-intensive	[127,128,129,130,131]
Self-assembly	Block copolymers (BCPs), such as P3HT, poly(n-butyl acrylate); peptide amphiphiles (PAs)	2–30 nm	- Complicated and layered frameworks - Adaptive and self-repairing characteristics - Streamlined processing - Enhanced recyclability - Accurate management of attributes - Excellent stability and longevity - Incorporation with living systems	- Absence of control - Unsteadiness - Complicated - Lengthy procedure	[150,151,152,153,154,155,156,157,158,159,160,161]

## 3. Applications of Nano- and Microfibers

Polymer fibers are utilized in actuating applications by transforming stimuli such as electrical, thermal, or chemical alterations into mechanical movement to generate artificial muscles for soft robotics, sensors, and various devices. Twisted, coiled, or intricate polymer fibers can shrink or extend when exposed to these stimuli, facilitating various uses, from basic grippers to intelligent fabrics [20,162]. Smart polymer fibers are classified based on their responsive behavior to stimuli, mainly comprising shape memory fibers (SMFs) that return to their original shapes, hydrogel fibers that expand or contract with moisture or pH changes, and liquid crystal fibers (LCFs) that alter their optical or physical properties, in addition to electroactive polymers (EAPs) for actuation, all reacting to heat, light, moisture, or electric fields for uses in textiles, sensors, and robotics [163].

### 3.1. Shape Memory Fibers (SMFs)

**Figure 4:**
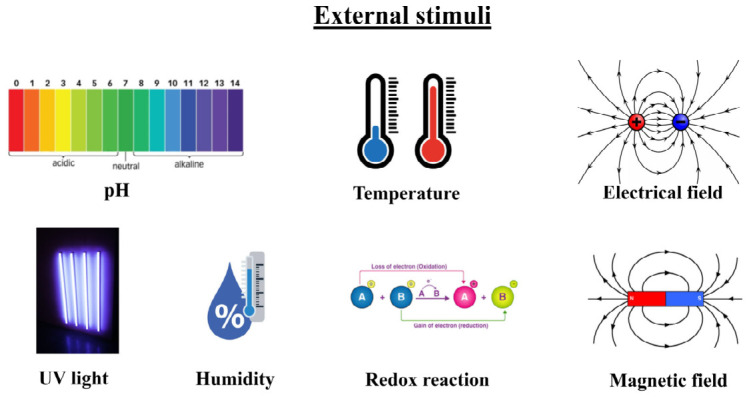
Activation stimuli for polymer fibers [166].

Polymer fibers can be engineered to be “stimulus-responsive”, meaning that they alter their characteristics when subjected to external factors such as temperature, light, humidity, or an electric field (Figure 4). As a result of these external stimuli, SMFs will be activated in different forms, including shrinking, expanding, or changing colors. They can be utilized for purposes like artificial muscles, sensors, and intelligent fabrics [164,165].

**Figure 5:**
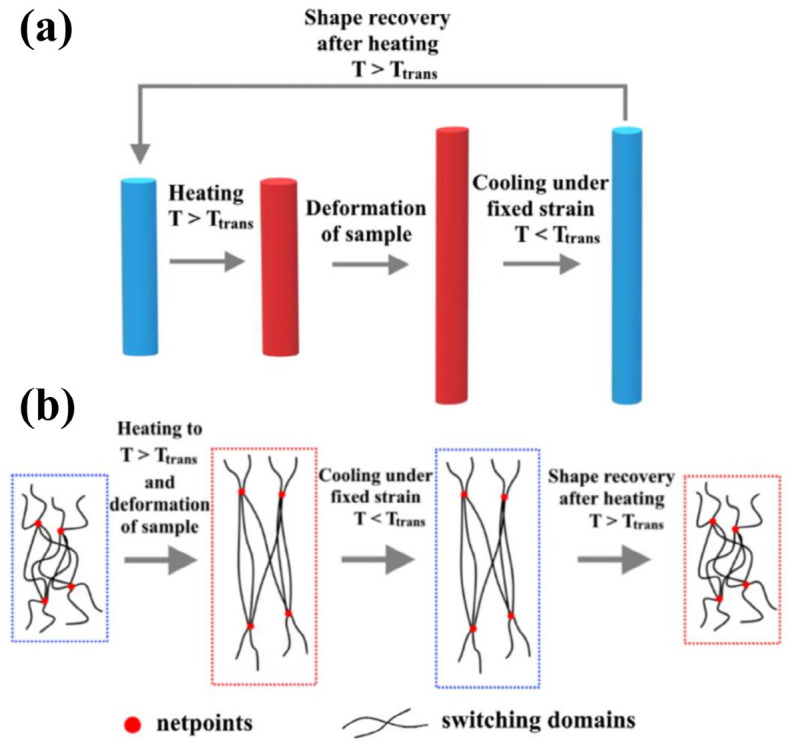
One-way SME in polymer fibers at macroscopic level (**a**) and at structure level (**b**) [170].

The most promising category of stimulus-responsive polymer fibers is the coiled type [20]. Typically, alterations in shape due to external factors, particularly heat, electric fields, and light, are linked to the molecular structure of the shape memory effect (SMP), characterized by netpoints and switching domains [167]. Figure 5 illustrates that netpoints can exhibit a physical nature, including entanglement coupling, crystalline phases, or copolymers, as well as a chemical nature, like covalent bonds; the polymer chains connecting the netpoints are referred to as switching domains. In the shape memory effect, the recollection of the original fixed form of the SMP is related to the netpoints, which dictate the permanent shape, while the reversible alteration of the SMP is associated with the switching domains, which manage the temporary shape [168,169]. The flexibility of polymer chains in the switching domains is associated with the transition temperature (T_trans_), which corresponds to the glass transition temperature (T_g_) for amorphous polymers or the melt temperature (T_m_) for semi-crystalline polymers [170]. By elevating the temperature of the SMP above T_trans_, the polymer chains within the switching domains gain significant flexibility, allowing deformation to achieve substantial strain with minimal stress. Subsequently, the polymer chains will become fixed, maintaining the temporary configuration when the polymer is cooled to a temperature below T_trans_, as shown in Figure 5. To revert to the permanent form, the polymer needs to be heated once more to a temperature exceeding T_trans_, enabling the switching domains to gain mobility and adopt a configuration that aligns with the permanent shape [168].

**Figure 6:**
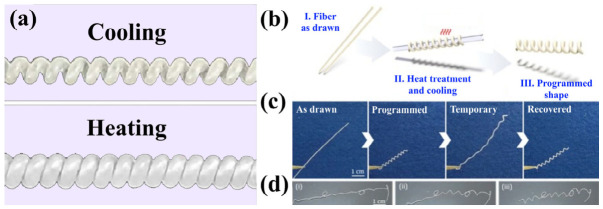
(**a**) Thermally activated coiled artificial material based on UHMWPE fibers [182]. (**b**) Programming process for a shape memory fiber: I—the fiber in its drawn state exhibits a linear form, II—the fiber is shaped over a template and heated to 190 °C for 1 min before being quickly cooled to ambient temperature, and III—3D illustration of the programmed form after the fiber is removed from the template. (**c**) Programming and shape recovery process of a shape memory fiber configured as a coil. (**d**) Shape-memory fiber (i) in a temporary state, (ii) transitioning, and (iii) in its intended shape, displayed in cursive memory [183].

The first report of coiled polymer fibers was in 2014, and they were made from nylon fishing lines [171]. This coiled polymer fiber could be simply fabricated by twisting and was activated by heat; it can be used in artificial muscles. Nowadays, numerous material and geometric designs have advanced the evolution of coiled polymer fibers for enhanced performance and a wider range of applications, including artificial muscles [172], twist fridges [173], electricity generators [174], smart hands [175], smart textiles [176], soft robotics [177,178], and sensors [179] (Table 2). Typically, polymer fibers with a semi-crystalline structure that are highly oriented result in anisotropic thermal expansion coefficients. This indicates that, as heating occurs, the crystalline areas of the polymer fibers increase in volume, whereas the amorphous polymer chains decrease in length because of the entropy elasticity of the polymer [180]. Consequently, the highly aligned fiber shortens in length while increasing in diameter. Additionally, torque stress is created due to the untwisting of coiled polymer fibers caused by thermal expansion. The torque stress may reduce intercoil separation and enhance the thermal contraction properties of coiled polymer fibers [181]. In [182], coiled artificial muscles utilizing aligned ultra-high-molecular-weight polyethylene (UHMWPE) fibers were developed using the extrusion technique. The authors showed that these actuators exhibited tensile stroke of up to 87% along with remarkable mechanical properties, as illustrated in Figure 6a.

In [183], the authors prepared a shape memory fiber through the preform-to-fiber drawing technique. Polylactic acid (PLA) was utilized as the polymer, exhibiting shape memory characteristics, while polyethylene terephthalate glycol (PETG) served as the amorphous coating to regulate the flow of molten PLA throughout the thermal elongation process; see Figure 6b–d. The authors indicated that fiber samples exhibited light transmission and were capable of changing from straight forms to curved forms, achieving angles of up to 170° and a bending radius of approximately 2.5 mm.

### 3.2. Hydrogel Fibers

Smart fibers in the form of hydrogels are innovative materials that combine sensing, energy storage, and actuation by utilizing their responsive characteristics (such as pH, temperature, and light) in a fibrous structure, allowing for use in wearable technology, soft robotics, and medical devices. They exhibit advantages like autonomous sensing, excellent conductivity, and flexibility, frequently including nanomaterials or conductive polymers to improve their functionality [184,185]. The primary features of hydrogel fibers include their classification as stimulus-responsive and biocompatible materials, and they have impressive mechanical attributes, such as high elasticity, stretchability, and toughness; additionally, they exhibit good conductivity [184,185]. Since hydrogel fibers are considered a three-dimensional network with the ability to adsorb water with structural stability, they are transforming several applications, such as functioning as smart reservoirs for water and nutrients in agriculture, encouraging tissue regeneration in healthcare, and acting as adaptive, responsive elements in materials science (Table 2).

These hydrogels can be widely used as smart materials. For example, in [184], the authors developed a hydrogel fiber material by employing ι-carrageenan molecular chains as the anionic structure, using Al^+3^ for crosslinking points, and allowing freely mobile K^+^ and Na^+^ ions to facilitate charge transport. They indicated that these hydrogels could be incorporated into externally powered or self-powered strain sensors or temperature sensors, with supercapacitor features and near-semiconductor properties. In [186], artificial muscles that were coiled and responsive to pH were created using aligned polyacrylonitrile (PAN) fibers, achieving contractive actuation stroke of 47%. The researchers demonstrated that the created artificial muscles exhibited a pH response by incorporating carboxyl groups into PAN fibers through a hydrolysis process following thermal stabilization.

### 3.3. Liquid Crystal Fibers

**Figure 7:**
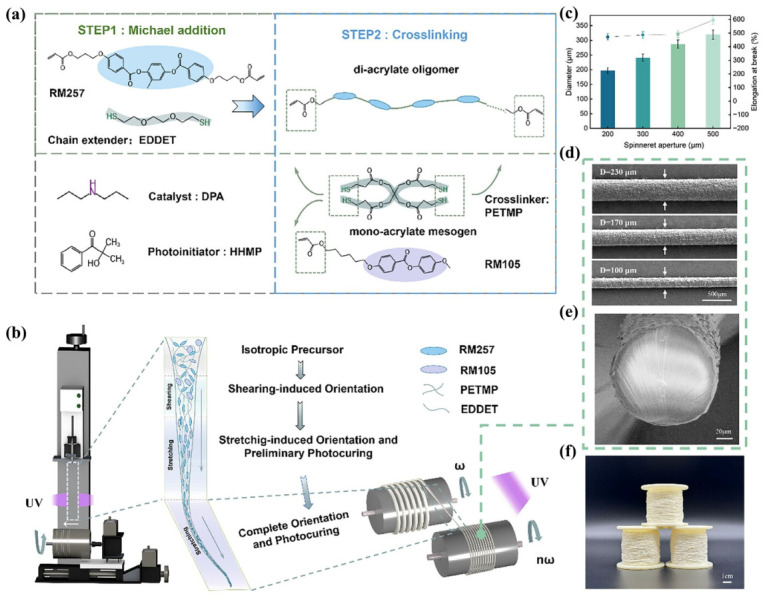
Production of mixed main-chain and side-chain LCE fibers through solution spinning. (**a**) Chemical formulation and reaction process of mixed main-chain and side-chain LCE fibers. (**b**) Diagram illustrating the two-step solution spinning method for LCE fiber creation. (**c**) Diameter and fracture elongation of polydomain LCE fibers produced with spinnerets of various aperture dimensions. (**d**) SEM images of monodomain LCE fibers exhibiting different diameters. (**e**) Cross-sectional SEM images depicting LCE fibers. (**f**) Image of LCE fibers wound on spools [193].

Liquid crystal elastomers (LCEs) are minimally crosslinked polymer networks that can undergo reversible deformation when exposed to external stimuli, such as thermal [187,188], optical [189], or electrical excitation [190,191]. The operational mechanism relies on the order–disorder phase transition of mesogenic unit alignment, wherein microscopic molecular shifts provoke macroscopic shape changes in the material, granting it excellent characteristics, such as rapid response times, substantial reversible deformation, and notable cyclic endurance [192]. This type of fiber also can be used in a wide range of applications, such as artificial muscles, soft robotics, drug delivery, environmental pollutant sensors, smart agriculture monitoring, high-strength fiber electronics, etc. (Table 2). The fibers exhibit a clear LCE morphology, distinguished by high aspect ratios and extensive specific surface areas. For example, in [193], the authors developed an LCE fiber that integrated both a main chain and side chains through the solution spinning technique (Figure 7). They reported that integrating monoacrylate-functionalized liquid crystal monomers to establish side-chain groups resulted in organized areas, demonstrating nematic–smectic phase separation within the macroscopically uniform elastomer. The produced LCE fibers could experience reversible deformation within an almost ambient temperature range of 35–65 °C and demonstrated reasonable actuation performance at up to 45%.

**Figure 8:**
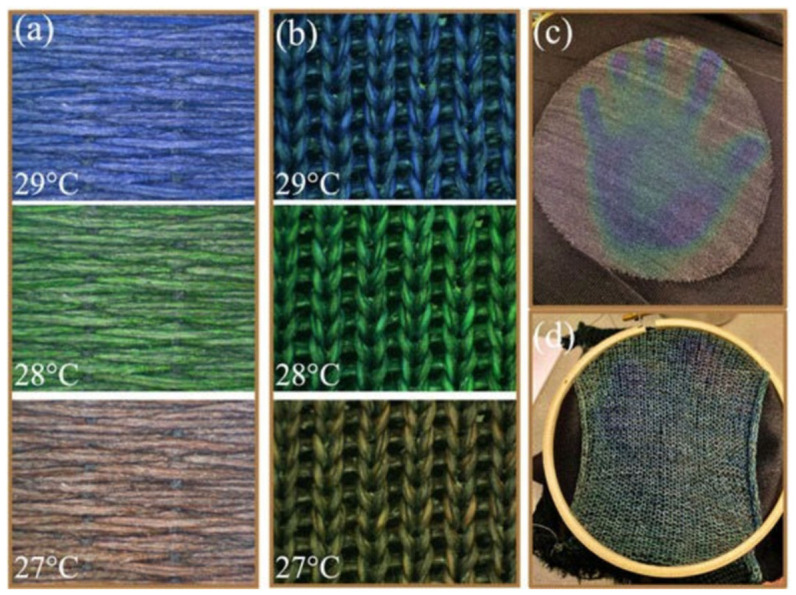
Pictures of handwoven (**a**) and knitted (**b**) textiles made from SFXC LC-coated polyester yarns. (**c**) Machine-knitted textile featuring a black satin fabric layer below, exposing the hand placed beneath it. (**d**) Handknitted textile demonstrating temperature irregularity [194].

**Figure 9:**
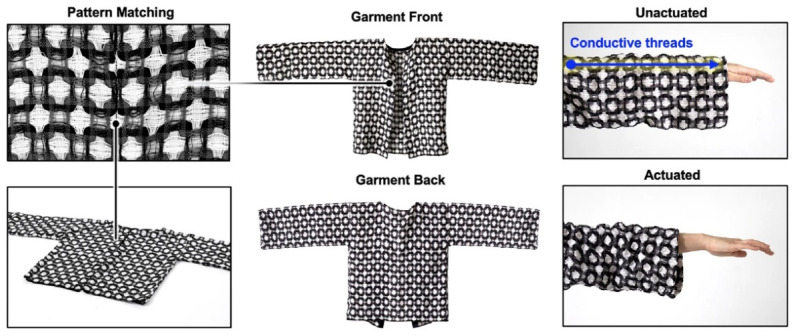
Summary of functional clothing prototype, outlining pattern alignment for accurate actuation, the front and rear of the clothing, and the internal thermal actuation (through resistive heating) of a single sleeve [195].

Agra-Kooijman et al., in their work [194], prepared a sample of breathable thermochromic fabric. They stated that their fabric was made from polyester filaments that were pre-coated with thermochromic liquid crystal (TLC) ink, instead of applying the TLC ink directly onto the woven/knitted textile. Furthermore, the authors demonstrated that samples from both knitted and handwoven materials exhibited outstanding reversible thermochromic properties, changing color from red to blue as the temperature increased from 26 to 32 °C, in alignment with the unincorporated TLC ink (Figure 8). In [195], the authors prepared handwoven robotic textiles based on several heat-responsive LCE fibers using the fiber extrusion method. To investigate the actuatable design space, they integrated their LCE fibers into a wide array of commercially significant woven textile structures, following a hierarchy in terms of the quantity of fabric layers. They demonstrated that, utilizing the capacity of single-layer materials to bend upward and revert to a flat surface, panels were successively actuated between temperatures of 25 and 65 °C (as recorded with a thermal camera); see Figure 9. The activation of thermo-responsive active fibers takes place over a spectrum of temperatures, starting at a lower temperature and concluding at a higher one. However, the temperature range differs based on the material used.

### 3.4. Electroactive Polymer Fibers

Electroactive polymer (EAP) fibers are intelligent materials that modify their shape/size through electrical stimulation, functioning as “artificial muscles” for lightweight, flexible actuators in soft robotics, prosthetics, and wearable devices. They imitate biological movement through electronic or ionic processes, providing significant strain but exhibiting issues with control and longevity, with a recent emphasis on fibrillary structures for enhanced performance [166,196]. EAPs are generally divided into electronic and ionic EAPs according to their activation method [197]. Electronic EAPs function via Coulombic forces produced by external electric fields, resulting in deformation with minimal ionic displacement [198]. Electronic EAPs demonstrate quick response times and function efficiently in arid conditions, making them ideal for aerospace, haptic interfaces, and rapid actuation systems [199]. Ionic EAPs undergo deformation from ion migration within the polymer matrix when exposed to low-voltage stimuli (<5 V), making them particularly effective in biomedical, soft robotics, and underwater applications because of their capacity to produce substantial bending displacements [200].

EAPs can be applied in different applications, such as artificial muscles, smart actuators, biosensors, engineering scaffolds, etc. (Table 2). From the point of view of actuation applications, electrothermal actuation is fundamental in creating artificial muscle fibers, skillfully transforming electrical energy into regulated thermal expansions and contractions to replicate the dynamic functions of natural muscles [201]. The process of electrothermal actuation entails transmitting an electrical current through an electrothermally conductive substance, resulting in Joule heating. The resulting rise in temperature leads to the expansion of the thermally active material found within the fibers. On the other hand, decreasing or stopping the electrical stimulation permits the fibers to cool and shrink, restoring them to their initial condition. An essential aspect of this mechanism is the inherent twisted arrangement of the fibers, which not only lengthen but also unwind during thermal expansion, thereby enhancing the actuation effect through a blend of axial stretching and rotational motion as they expand in volume. Consequently, the substances quickly produce Joule heat and evenly distribute this heat to the matrix material. This process triggers thermal expansion, which is subsequently transformed into actuation behavior in synthetic muscle fibers [202,203]. In the case of coatings, this outer layer functions as a resistive heater, allowing the electrical current to pass through the coating and produce Joule heat [204,205]. The heat is subsequently directed to the underlying polymer, leading to its expansion and activation.

In [202], by applying the technique that spiders use to manage uncontrolled spinning in dragline silk, the authors created ultrafast hybrid carbon nanotube yarn muscles that achieved rotation of 9800 rpm with minimal oscillation. A high-loss viscoelastic substance, consisting of paraffin wax and a polystyrene–poly(ethylene-butylene)–polystyrene copolymer, served as the yarn guest to provide an overdamped dynamic reaction. They demonstrated dynamic mirror positioning that was both rapid and precise, and there was a more than 10-fold reduction in the mechanical stabilization time when compared to earlier torsional muscles derived from nanotube yarn. Scalability to maintain a consistent volumetric torsional work capacity was shown across a 10-fold variation in the yarn cross-sectional area.

**Figure 10:**
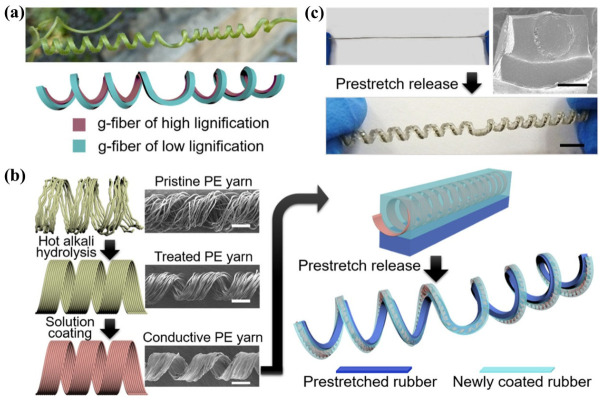
The design and creation of a biomimetic conductive tendril. (**a**) The curling model of a cucumber tendril. (**b**) The diagrammatic representation of the manufacturing process of the conductive tendril. Scale bar for SEM images: 400 μm. (**c**) Images of the conductive tendril prior to (upper) and after (lower) prestretch release. The SEM image in the upper right displays the cross-section of the conductive tendril. Scale bar for photo of conductive tendril: 4 mm. Scale bar for SEM image: 400 μm [206].

In [206], a conductive tendril was created to replicate the functional advantages of plant tendrils using PE yarn (Figure 10). The authors demonstrated that the tendril configuration allowed for the significant extension of the stretching range from strains of 225% to 2000%, with a minimal resistance rise of 9.2%, making it suitable for applications in ultrastretchable electronics, including ultrastretchable LED lighting and earphone audio cables. Additionally, the conductive tendril demonstrated outstanding actuation capabilities—achieving contraction stroke of 19.8% or lifting 120 times its weight. Furthermore, the authors indicated that their fibers, as artificial muscles, could function autonomously as an elastic dragger and achieve complex programmable movement in a cohesive way, similar to a smart gripper.

Meanwhile, in [204], the authors provided an example of coated polymer fibers. They created a twisted and coiled soft actuator (STCA) using spandex fibers. This STCA showed greater actuation strain at reduced temperatures compared to earlier nylon twisted and coiled soft actuators (NTCAs). They stated that, while the NTCAs were created through a twist insertion method until coils were established, a novel approach was introduced to manufacture the STCA using the ultra-stretching of spandex, in which the STCA was twisted once more after the coil had been established. Outstanding performance was demonstrated for the STCA, resulting from the 14% contraction strain of the bare spandex (bare nylon: 4%) and the low spring constant of 0.0115 N mm^−1^. The highest tensile actuation strain of STCA reached 45% at 130 °C, and the peak specific work was 1.523 kJ kg^−1^ at 130 °C. The STCA could actuate 100 times in a row with a strain variation of under 0.4%. Another example is provided in [205], where the authors prepared artificial muscles that were reported to be based on highly aligned nylon and polyethylene fibers. These synthetic muscles could be triggered by twisting and coiling monofilaments made from these substances to create helical springs, allowing for anisotropic thermal expansion; this facilitated tensile actuation of up to 49% when heated by applying a current to a conductive layer on the filament’s surface. The authors indicated that the formulated materials exhibited up to 29% linear actuation, with energy and power densities of 840 kJ m^−3^ (528 J kg^−1^) and 1.1 kW kg^−1^ (functioning at 0.1 Hz, 4% strain, and 1.4 kg load).

**Table 2:** Applications of polymeric fibers.

Fiber Type	Applications	References
Shape memory fibers (SMFs)	- Smart greenhouse fabrics - Tissue engineering scaffolds - Self-tightening sutures - Compression garments - Responsive textiles - Creaseless fabrics - Protective clothing for agricultural workers	[207,208,209,210,211]
Hydrogel fibers	- Soil amendment - Water retention - Precision agriculture - Drug delivery - Soft actuators and sensors - Smart wound patches - Conductive materials	[212,213,214,215]
Liquid crystal fibers	- Artificial muscles - Soft robotics - Smart textiles for health monitoring - Drug delivery - Environmental pollutant sensors - Smart agriculture monitoring - High-strength fiber electronics - Mechanochromic sensors	[194,216,217,218,219,220,221]
Electroactive polymer fibers	- Artificial muscles - Tissue engineering scaffolds - Biosensors - Soft actuators - Biocompatible and biodegradable actuators	[162,222,223,224,225]

## 4. Future Prospects

The development of low-cost, scalable, and electrically conductive polymer fibers derived from thermoplastics remains a critical challenge for next-generation actuation systems. Achieving high electrical conductivity without compromising mechanical integrity is particularly difficult due to limitations in maintaining stable percolation networks of conductive fillers within polymer matrices. Future research should therefore focus on optimizing fabrication strategies that enable the large-scale production of affordable, mechanically robust, and electrically conductive fibers. In parallel, advancing shape memory polymer fibers beyond one-way actuation toward reliable and efficient two-way reversible systems remains a significant scientific and engineering challenge. Reducing recovery times, improving cycling stability, and achieving precise control over actuation behavior will require a deeper understanding of polymer chemistry, molecular architectures, and filler–matrix interactions. Addressing these challenges will be essential in translating smart polymer fiber technologies from laboratory demonstrations to practical, real-world applications.

Data-driven technologies and artificial intelligence (AI) are fundamentally revolutionizing the design, production, and usage of polymer fibers for intelligent actuation, moving the domain from experimental trial and error to predictive, automatic, and high-efficiency engineering. These technologies enable the enhancement of intricate polymer fiber actuators—like twisted and coiled polymer muscles, liquid crystal elastomers (LCEs), and electroactive polymers (EAPs)—by examining extensive datasets to forecast performance and streamline synthesis [226,227]. For instance, machine learning (ML) models like neural networks are educated using experimental data to forecast essential characteristics of polymer actuators, including strain, stress, actuation speed, and lifespan, without extensive testing. Generative AI allows researchers to specify preferred actuation characteristics (e.g., particular contraction at a low voltage) and to design the polymer’s molecular structure inversely to achieve these goals. Additionally, AI enhances fabrication methods such as electrospinning by fine-tuning parameters in real time, minimizing material flaws and boosting uniformity. Conversely, methods like Gaussian process regression, support vector machines, and random forest are frequently employed to develop structure–performance maps for intelligent fibers. Active learning and Bayesian optimization techniques are essential in handling sparse data situations, determining the most effective subsequent experiments, and fine-tuning parameters in intricate systems such as coiled polymer actuators [228,229].

## 5. Conclusions

Polymer fibers play a pivotal role in modern materials engineering owing to their unique combination of a light weight, high strength, flexibility, and durability. These attributes have enabled their widespread adoption across diverse sectors, ranging from textiles (e.g., bulletproof vests and apparel) to aerospace (lightweight composite structures) and construction (fiber-reinforced concrete). Their ability to replace heavier and corrosion-prone materials such as steel and asbestos offers significant advantages in terms of cost reduction, performance enhancement, and sustainability, while also supporting advanced applications in medical devices, automotive components, and robotics.

This review highlights that electrospinning and melt blowing remain the most extensively utilized techniques for the fabrication of nano- and microscale polymer fibers. Electrospinning is particularly attractive due to its high surface-area-to-volume ratio, tunable fiber properties, versatility, low cost, and simple setup. It enables the production of ultrafine nanofibers from polymer solutions under high electric fields, offering excellent control over the fiber morphology. However, its relatively low production rate and reliance on solvents limit large-scale manufacturing, confining its use primarily to high-value applications such as biomedical scaffolds and high-efficiency filtration. In contrast, melt blowing employs high-velocity hot air to attenuate molten polymers, allowing for the high-throughput, industrial-scale production of microfibers. While this method is well suited for bulk applications such as filtration media, it offers less precision at the nanoscale and involves higher thermal constraints.

Solution blowing provides an intermediate approach, enabling the formation of finer and more uniform submicron or nanofibers with improved controllability compared to melt blowing, albeit at the expense of greater process complexity and solvent-related costs. Dry and wet spinning techniques further expand the fabrication landscape: dry spinning relies on solvent evaporation via hot air, allowing faster processing and lower operational costs, whereas wet spinning utilizes a coagulation bath to solidify polymer solutions, offering superior control over the fiber morphology, porosity, and mechanical strength, albeit with reduced production rates and more extensive post-processing requirements.

This review also highlights the growing importance of polymer fibers in actuation applications. Smart polymer fibers provide diverse actuation pathways, including shape memory polymers for large, thermally driven shape transformations in aerospace and structural systems; electroactive polymers for rapid and electrically controlled actuation in robotics; and hydrogel fibers that excel in high-strain and high-sensitivity responses for wearable sensors. Key performance metrics such as the actuation mechanisms, response times, strain and force output, energy efficiency, and environmental sensitivity vary significantly among these systems. Hybrid architectures, including coiled fibers and fiber-reinforced composites, frequently outperform single-material fibers by enhancing the load capacity, actuation speed, and durability.

## Data Availability

The data presented in this study are available on request from the corresponding author.

## Abbreviations

The following abbreviations are used in this manuscript:

PET	Polyethyleneterephthalate
PAN	Polyacrylonitrile
PTFE	Polytetrafluoroethylene
PVA	Polyvinyl alcohol
PCL	Polycaprolactone
PVDF	Polyvinylidene fluoride
PP	Polypropylene
PET	Polyester
PE	Polyethylene
UHMWPE	Ultra-high molecular weight polyethylene
PEO	Poly(ethylene oxide)
EVA	Ethylene-vinyl acetate
PVP	Poly(vinyl pyrrolidone)
PLA	Polylactic acid
PU	Polyurethane
PVC	Polyvinyl chloride
CA	Cellulose acetate
PMMA	Poly(methyl methacrylate)
PANI	Polyaniline
PPy	Polypyrrole
PLGA	Poly(lactic-co-glycolic acid)
PGA	Poly(glycolic acid)
PAAM	Polyacrylamide
PEEK	Polyetheretherketone
BCP	Block copolymer
P3HT	Poly(3-hexylthiophene)
PAs	Peptide amphiphiles
SMF	Shape memory fiber
LCE	Liquid crystal elastomer
EAP	Electroactive polymer
DC	Direct current
AC	Alternating current
TIPS	Thermal induced phase separation
VIPS	Vapor-induced phase separation
NIPS	Non-solvent-induced phase separation
AI	Artificial intelligence
ML	Machine learning
